# Modeling manufacturing resources based on manufacturability features

**DOI:** 10.1038/s41598-022-15072-2

**Published:** 2022-06-24

**Authors:** Changlong Zhao, Chen Ma, Haifeng Zhang, Zhenrong Ma, Junbao Yang, Ming Li, Xuxu Wang, Qiyin Lv

**Affiliations:** College of Mechanical and Vehicle Engineering, Changchun University, Changchun, 130022 China

**Keywords:** Mechanical engineering, Computer science

## Abstract

Manufacturability evaluation is an effective way to shorten the development period, optimize manufacturing processes, and reduce product costs. The manufacturability of a product depends on the processing ability of specific manufacturing resources. The development of a manufacturing resources model serves as the foundation for manufacturability evaluation. To better utilize the information on manufacturing resources, this study adopted a hybrid approach by integrating the fuzzy c-means clustering algorithm and the genetic algorithm to group manufacturing resources based on manufacturing and geometric features. The information model of manufacturing resources was built by using the object-oriented method. Subsequently, the framework to evaluate manufacturing capability based on manufacturing resources was defined. Further, an application sample was exploited and its results were analyzed. The results of the subgroup showed that the hybrid algorithm was reliable and valid and helped improve the overall performance of the company chosen for this study. The proposed approach enhanced feasibility in decision-making and facilitated the management to make more informed decisions.

## Introduction

In Industry 4.0, smart manufacturing has become the development direction in manufacturing industries globally^1^. The increasingly fierce global competition has posed challenges to the manufacturing industry, the most significant of which is the problem of unbalanced data sets. Leng et al. (2021) developed a loosely coupled integration model based on deep learning and reinforcement learning techniques in the context of Industry 4.0^2^ and employed granularity-based computing^3^ and deep learning^4^ innovative models to successfully refine unbalanced datasets in manufacturing.

To design it right the very first time, designers must ensure that their products are both functional and easy to manufacture. A manufacturability analysis system^5–7^ can be used to evaluate various manufacturability aspects in the design phase, thereby reducing the cost and time to market of the designed product and promoting the development of virtual manufacturing. The manufacturability evaluation of a proposed design involves determining whether or not a product can be manufactured by using the available manufacturing resources and, if so, finding the associated manufacturing efficiency. A product can be manufactured quickly by ensuring low cost and high quality in some manufacturing settings; however, in other facilities, the manufacturing costs can be high, even leading to situations where the product cannot be manufactured. In such manufacturing environments, by using the existing resources, a product can be manufactured with low cost and high efficiency by using different equipment. Manufacturing resources not only aid production design, process design, and manufacturing but also impose restrictions. Thus, it is crucial to build a robust manufacturing resources model for manufacturability evaluation. The process of building this model comprises two parts: grouping of processing equipment and information modeling of manufacturing resources.

The concept of manufacturing resources can be viewed from both narrow and broad senses. From a broad viewpoint, manufacturing resources involve all the elements required that are connected with the design, processing, maintenance, and other processes in the entire product life cycle; from a narrow sense, however, manufacturing resources involve the equipment, cutting tools, materials, fixtures, measures, and other implements that are only connected with processing. Information about manufacturing resources not only provides support for product design, process design, and manufacturing but also places constraints on these processes. Clustering of processing equipment reduces the searching space and time taken for manufacturability evaluation. Manufacturing resources can thus be utilized more efficiently. The aim of the clustering method is to organize a group of objects into classes or clusters such that objects belonging to the same cluster are similar enough to infer that they are of the same type; meanwhile, objects belonging to different clusters will be similar enough to infer that they are of different types^8^.

With the rapid pace of industrial development, many new intelligent manufacturing techniques have emerged, such as digital twin technology^9,10^, which uses a computer program to achieve interaction between virtual and actual processing, whereby the virtual control of the actual processes can reduce costs and improve production efficiency. However, the application of digital twin technology to actual production processes calls for the selection of manufacturing features that depend on the processing equipment. As the processing capabilities of modern equipment become larger, the processing capacity between different equipment becomes increasingly fuzzy. Thus, to improve the actual production efficiency, this paper proposes a hybrid algorithm, combining the genetic algorithm (GA) and fuzzy c-mean (FCM) clustering algorithm based on GA, to group manufacturing resources according to their processable characteristics. The hybrid algorithm possesses both the global searching ability of GA and the local searching ability of the fuzzy clustering algorithm. This way, the optimum number of clusters and optimum partitions can be obtained simultaneously, which reduces the searching time and searching space for proper processing equipment. The information model of manufacturing resources was built by using the object-oriented method on the basis of analyzing the related information required for processing manufacturing resources. This study also incorporated features such as hole, plane, and step in the manufacturing resources model. Finally, a framework for evaluating manufacturability based on manufacturing resources constraints was devised.

## Feature-based grouping of processing equipment

Manufacturability evaluation^11,12^ is directly linked to manufacturing resources. A product can be manufactured quickly by ensuring low cost and high quality in some manufacturing settings; however, in other facilities, the manufacturing costs can be high, even leading to situations where the product cannot be manufactured. In such manufacturing environments, by using the existing resources, a product can be manufactured with low cost and high efficiency by using different equipment. If the constraints of manufacturing resources are not considered, manufacturability evaluation does not make sense. A modern production firm would always have rich processing equipment. To utilize them better, the efficiency of manufacturability evaluation needs to be increased and the processing equipment has to be partitioned. There are different principles of partition. With the development of feature technology, feature-based manufacturability evaluation has turned into a research hotspot. These features include economic indicators, technical indicators, productivity indicators, and environmental indicators. Different manufacturability evaluation methods have been proposed based on different features^13^. The feature-based grouping of processing equipment makes manufacturability evolution more conducive.

Manufacturing processes can include many features, such as plane, hole, blind hole, step, slot, blind slot, pocket, cylindrical protrusion, and curved surface. Processing equipment is grouped according to the features that can be processed by the equipment. However, the same features cannot always be processed by the same equipment because of varying part sizes, tolerance requirements, and other important manufacturing criteria. In this study, in addition to the features, the part size and processing accuracy were also considered as processing equipment attributes. In feature-based manufacturing resources partition, there are N processing equipment and s features altogether. The processing equipment vector can be defined through Eqs. (1) and (2).

1 $$x_{i} = \left( {x_{i1} ,x_{i2} , \ldots ,x_{is} ,p_{i} ,a_{i} } \right)\quad i = 1,2, \ldots ,N$$

2 $$\begin{gathered} x_{ik} = \left\{ \begin{gathered} 1\quad {\text{equipment}}\;{\text{i}}\;{\text{can}}\;{\text{process}}\;{\text{feature}}\;{\text{k}} \hfill \\ 0\quad {\text{equipment}}\;{\text{i}}\;{\text{can}}\;{\text{not}}\;{\text{process}}\;{\text{feature}}\;{\text{k}} \hfill \\ \end{gathered} \right. \, k = 1,2, \cdots ,s \hfill \\ p_{i} = \left\{ \begin{gathered} 1\quad {\text{equipment}}\;{\text{i}}\;{\text{can}}\;{\text{process}}\;{\text{large - sized}}\;{\text{parts}} \hfill \\ 0\quad {\text{equipment}}\;{\text{i}}\;{\text{only}}\;{\text{can}}\;{\text{process}}\;{\text{small}}\;{\text{and}}\;{\text{medium}}\;{\text{parts }} \hfill \\ \end{gathered} \right. \hfill \\ a_{i} = \left\{ \begin{gathered} 1\quad {\text{equipment}}\;{\text{i}}\;{\text{can}}\;{\text{be}}\;{\text{used}}\;{\text{in}}\;{\text{finish}}\;{\text{machining}} \hfill \\ 0\quad {\text{equipment}}\;{\text{i}}\;{\text{can}}\;{\text{not}}\;{\text{be}}\;{\text{used}}\;{\text{in}}\;{\text{finish}}\;{\text{machining}} \hfill \\ \end{gathered} \right. \, \hfill \\ \end{gathered}$$

**Figure 1:**
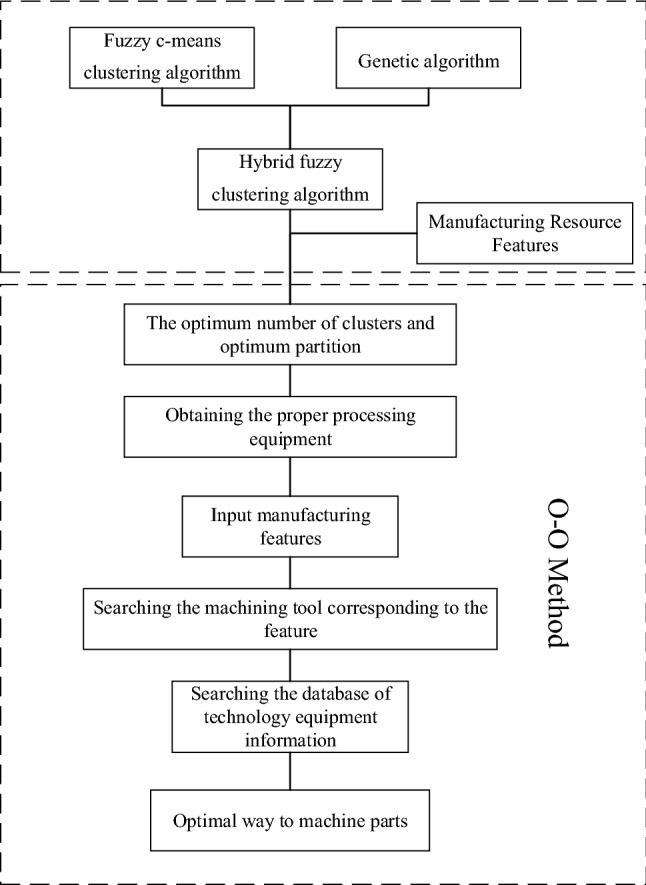
Overall flow chart of manufacturing resources modeling.

There are a few clustering algorithms, and the FCM algorithm can be used to partition the manufacturing resources here. The overall process of manufacturing resource modeling is shown in Fig. 1.

## Hybrid algorithm of FCM and GA for grouping manufacturing resources

### Fuzzy c-means (FCM) algorithm

The FCM clustering algorithm is an unsupervised and non-parametric method that can help to analyze data through cluster analysis. The technique was first proposed in 1973 and has been widely used since. The FCM has been proven to have good stability and partition quality through some cases and good convergence^14^. An FCM convergence proof can be established as follows^15–18^.

Given a set of objects $X = \left( {x_{1} ,x_{2} , \ldots ,x_{N} } \right),x_{i} \in R^{s}$, where *N* is the number of objects and s is the dimension of the pattern vectors, FCM can be used to divide the region and find the optimal corresponding partition and prototype to minimize the following objective function.

3 $$J_{m} \left( {U,V} \right) = \sum\limits_{j = 1}^{C} {\sum\limits_{i = 1}^{N} {u_{ij}^{m} } } d_{ij}^{2}$$

where *C* is the number of clusters, *U* is the matrix of membership functions, $\mu_{ij}$ is the element of *U* and the membership value of the *i*th object of the *j*th cluster , *V* is the clustering center vector, m is the index controlling the amount of $\mu_{ij}$ cluster overlap and fuzziness,$d_{ij} = \left\| {\left. {x_{i} - v_{j}^{\left( t \right)} } \right\|} \right.$ represents the distance between *x*_*i*_ and *v*_*j*_, and *t* denotes the *t*th iteration.

The standard Lagrange multiplier minimization method can be invoked in Eq. (3) to obtain the updated clustering centroid vector and membership function matrix.

Given a fixed number *C* ($2 \le C < N$), *m* ($1 < m < \infty$) and $\varepsilon$ (a small positive constant), the FCM algorithm starts with a set of initial cluster centers. It then randomly generates a fuzzy c-partition and sets the iteration number $t = 0$, $t = \left( {0,1,2, \cdots \cdots } \right)$. The three-step iterative process works as follows:

**Step 1.** Given the membership values $\mu_{ij}^{\left( t \right)}$, the cluster center vector V is calculated by

4 $$v_{j}^{\left( t \right)} = \frac{{\sum\nolimits_{i = 1}^{N} {\left( {\mu_{ij}^{{\left( {t - 1} \right)}} } \right)^{m} } x_{i} }}{{\sum\nolimits_{i = 1}^{N} {\left( {\mu_{ij}^{{\left( {t - 1} \right)}} } \right)^{m} } }}\quad \, j = 1, \ldots C$$

**Step 2.** Given the new cluster centers $V^{\left( t \right)}$, the membership values $\mu_{ij}^{\left( t \right)}$ can be updated as

5 $$\mu_{ij} = \frac{1}{{\sum\nolimits_{k = 1}^{C} {\left( {\frac{{d_{ij} }}{{d_{ik} }}} \right)^{{\frac{2}{{\left( {m - 1} \right)}}}} } }}\quad i = 1, \ldots ,N\; \, j = 1, \ldots C$$

**Step 3.** Compare $U^{\left( t \right)}$ to $U^{{\left( {t + 1} \right)}}$ in a convenient matrix norm: if $\left\| {U^{{\left( {t + 1} \right)}} - U^{\left( t \right)} } \right\| < \varepsilon$, then stop; otherwise, set $t = t + 1$ and return to step 1.

### The genetic algorithm (GA)

In 1975, inspired by the evolutionist theory explaining the origin of species, Professor J. Holland proposed the GA. In nature, weaker species become extinct by natural selection while stronger species emerge from natural selection to pass on their genes to future generations. In the long run, dominant populations tend to be the species that carry the correct combination of genes. During the slow evolution of a species, genes can change at any time. If these changes are aided by natural selection, a new species is formed; conversely, if these changes do not aid natural selection, it eliminates any unsuccessful changes as the challenge to survive continues to increase. Because GA replaces many computationally expensive deterministic optimization methods, it has gained increasing popularity in the engineering field^19–21^.

### The hybrid fuzzy clustering algorithm

Since the FCM algorithm is a local search algorithm, it is effective in some areas; however, overall, its performance fails to meet people’s expectations^22^. The algorithm is particularly sensitive to initialization, leading to easy access to local optima during computation^23^. The GA is a global optimization algorithm widely used in practice and has advantages because of its universality and simplicity. It also has good robustness and fitness for concurrent processing. Based on these advantages, an FCM algorithm based on GA not only offers the GA’s global searching ability but also the local searching ability of FCM. The hybrid algorithm can solve the issue of the FCM algorithm being sensitive to initialization and increase the convergence speed. Consequently, clustering can be done more efficiently.

With the FCM algorithm, it is difficult to determine the number of initial clusters without effective guidance before performing clustering. The hybrid fuzzy clustering algorithm comprises outer and inner iterations. The outer iteration determines the optimal number of clusters dynamically by using the GA, and the inner iteration determines the optimal partition corresponding to the optimal number of clusters by using FCM clustering based on the GA.

### Inner iteration

Since the hybrid algorithm of GA and FCM is introduced in the internal iteration, the optimal classification matrix corresponding to the number of clusters can be easily obtained according to the principle of maximum membership. The main functions of the hybrid algorithm include encoding, constructing the fitness function, selecting the genetic operators, and determining the parameters^24^.

#### Encoding

Real coding on the clustering center *v* can be performed by the coding method^25^. A chromosome is expressed as $chr = v_{1} v_{2} \cdots v_{c}$,$v_{i} \left( {i = 1,2, \cdots ,c} \right)$, where C is the number of clusters. There are S characters in each cluster, so the length of a chromosome is $c \times s$. A chromosome is expressed as {v_11_,v_12_,...,v_1s_,v_21_,v_22_,v_2s_,......,v_c1_,v_c2_,...,v_cs_}.

#### Fitness function

The purpose of fuzzy clustering is to obtain the minimum objective function (loss function), which constitutes an optimization problem^26^. The method of determining the chromosomal fitness value for the survival probability of the next generation of an individual is a vital issue during optimization. The objective function of fuzzy clustering ($J_{m}$) should be small and the partition should be more reasonable. The corresponding fitness function of GA should also be big. The fitness function with the objective function $J_{m}$ can be defined as follows:

6 $$F\left( {U,V} \right) = \frac{1}{{J_{m} + \varepsilon }} \, \varepsilon > 0$$

#### Crossover and mutation operator

The most important operator in the GA is the crossover operator. An offspring is produced during the crossover process, which is defined as two chromosomes from the parents joining together to form a new chromosome. Upon iterating the crossover operator, the expected good chromosome genes appear frequently in the population, leading to convergence and an overall good solution. The double-point crossover operator is also employed. The two-point crossover operator involves a random selection of two crossover points, and fragments corresponding to the crossover points on two parental genes are exchanged^27–29^.

The variation operator plays a key role in the GA by introducing stochastic changes during chromosome evolution. The crossover uses an iterative approach to make the chromosomes in a population similar and thus converge the population, whereas mutation introduces random variation into the population and helps in searching, avoiding local optima. Because the mutation rate is very small, the new chromosomes created by mutation are not very different.

#### Selection operator

Simple GAs do not guarantee convergence of results to the global optimum solution. However, the GA when applied with the optimum individual maintaining strategy can yield a global optimum solution^30–32^. Thus, for this hybrid algorithm in this study, the selection was carried out by combining the remainder stochastic sampling with replacement and the optimum individual maintaining strategy. The advantage of remainder stochastic sampling with replacement is that individuals with high fitness can be preserved during child generation with a minimum selection error. Individuals with the biggest value of fitness function are maintained in the offspring without genetic manipulation.

#### FCM optimization of individuals

Due to the greater local searching capability of FCM, the population can be optimized using FCM after each generation of genetic manipulation to generate new populations for subsequent evolution. Using the FCM optimization method, the convergence speed can be improved and the local searching capability can be enhanced^33^. The realization of FCM optimization can be performed as follows:

1. The corresponding fuzzy matrix *U* is derived by calculating the chromosome code through Eq. (5).
2. The new clustering matrix *U* is calculated by Eq. (4) to derive the new clustering centers, which are encoded to generate new chromosomes.
3. By recalculating the value of the objective function, the worst individual in the population is found and is replaced with an individual that always remained the best during selection.

### Outer iteration

The FCM algorithm must determine the number of clusters in advance, and the process cannot be optimized. A typical GA uses outer iterations to determine the optimal number of clusters. A good clustering algorithm takes into account both the degree of separation between the different partitions and the degree of compression of a partition. The degree of dispersion between the different partitions can be expressed as the average distance between the clustering centers. The bigger the value of the average distance between the cluster centers, the greater the degree of deviation of the different partitions. The distance between the clustering centers is denoted by *D* and is expressed as follows:

7 $$D = \frac{{\sum\limits_{i = 1}^{C} {\left\| {v_{i} - v_{j} } \right\|} }}{C}$$

The main purpose of clustering is to partition the dataset in such a way that the distance between the different partitions is maximized and the distance between each object in a cluster is minimized. As the number of clusters (C) increases, the value of *J*_*m*_ decreases and the value of *D* increases. The objective function of outer iteration can be defined as follows:

8 $$f = J_{m} \left( {U,V} \right) + D$$

The fitness function of the outer iteration is defined as

9 $$F^{\prime}\left( {U,V} \right) = \frac{1}{{J_{m} \left( {U,V} \right) + D}}$$

The encoding method in a typical GA is a binary encoding of the number of clusters. The hybrid of optimum individual maintaining strategy and remainder stochastic sampling with replacement is employed as the selection operator. The crossover and mutation operators are the single-point crossover and essential mutation, respectively. The number of clusters corresponding to each chromosome is calculated, and the corresponding optimum partition is obtained by using the inner iteration.

## Implementation of algorithm

**Figure 2:**
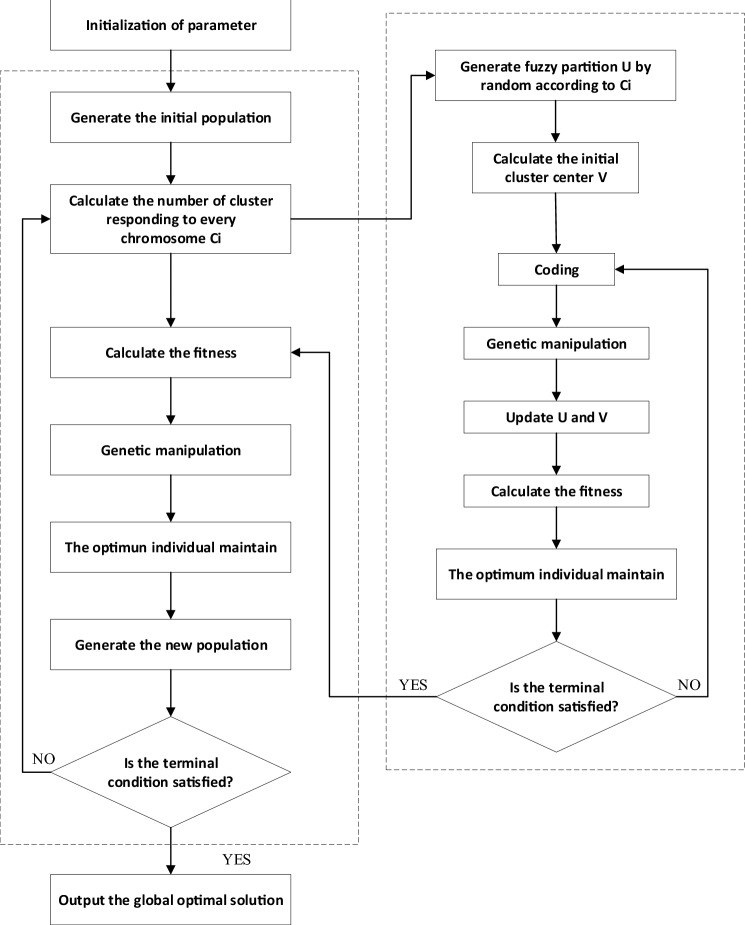
Schematic representation of the hybrid approach.

The hybrid algorithm that introduces manufacturing resource division comprises two parts: outer iteration and inner iteration. The program flow chart is shown in Fig. 2.

**Figure 3:**
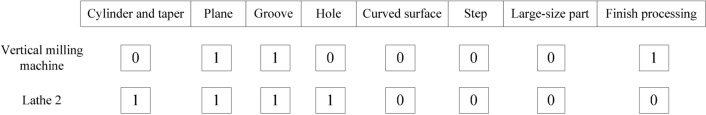
Vector representation of manufacturing resource.

To test the effectiveness of the algorithm, the set of manufacturing resources shown in Table 1 was divided based on the features that can be processed by the device. The relevant features considered by this study were cylinders and tapers, planes, bevels, holes, surfaces, and steps. The device was represented by a pattern vector, and the length of the pattern vector was 8. The first six digits represent cylinders, tapers, planes, grooves, holes, surfaces, and steps. The two diagrams on the left indicate the dimensions of the parts that can be machined with machining equipment and whether the machining equipment can be used for finishing. The mode vector comprised 0 and 1, corresponding to a value of 1 if the machine can handle the feature and 0 otherwise. Similarly, 1 if the machine can process large parts and 0 otherwise and 1 if the machine can be used for finishing and 0 otherwise. For example, the vertical milling machine can handle a plane slot but cannot handle a large part and can be used for finishing. Thus, the vector is 01100001. Lathe 2 can handle cylinder and cone, plane, slot, and hole but cannot handle a large part and can be used for finishing. Thus, the vector is 1,110,000, as shown in Fig. 3. The pattern vectors of 32 machining equipment are shown in Table 1. The equipment was grouped using the algorithm proposed by this study.

**Table 1:** A set of manufacturing resources.

Number	Device name	Features	Pattern vector
1	Vertical milling machine	Plane, groove, finish processing	011000 01
2	Drilling machine 1	Hole	000100 00
3	Drilling machine 2	Hole	000100 00
4	Drilling machine 3	Hole, large-size part	000100 10
5	Lathe 1	Cylinder and taper, hole	100,100 00
6	Lathe 2	Cylinder and taper, plane, groove, hole	111,100 00
7	Lathe 3	Cylinder and taper, plane, groove, hole, curved surface, step, finish processing	111,111 01
8	Lathe 4	Cylinder and taper, hole, finish processing	100,100 01
9	Lathe 5	Cylinder and taper, hole	100,100 00
10	Lathe 6	Cylinder and taper, plane, groove, hole, large-size part, finish processing	111,100 11
11	Drilling machine 4	Hole	000100 00
12	Milling and drilling machine	Plane and hole	010100 00
13	Drilling machine 5	Hole	000100 00
14	Boring-milling machine 1	Cylinder and taper, plane, hole, large-size part, finish processing	110,100 11
15	Coordinate setting boring machine	Plane, groove, hole, large-size part, finish processing	011100 11
16	Boring-milling machine 2	Plane, groove, hole, large-size part, finish processing	011100 11
17	Horizontal fine-boring machine	Hole, finish processing	000100 01
18	Milling machine 1	Plane, groove, finish processing	011000 01
19	Milling machine 2	Plane, curved surface, large-size part, finish processing	010010 11
20	Milling machine 3	Plane, groove, step, finish processing	011001 01
21	Milling machine 4	Plane, groove, curved surface, step, finish processing	011011 01
22	Milling machine 5	Plane, groove, hole, step, finish processing	011101 01
23	Milling machine 6	Plane, large-size part, finish processing	010000 11
24	Milling machine 7	Plane, large-size part, finish processing	010000 11
25	Milling machine 8	Plane, curved surface, large-size part, finish processing	010010 11
26	Boring-milling machine 3	Plane, hole, large-size part, finish processing	010100 11
27	Planning machine 1	Plane, groove	011000 00
28	Cylindrical grinder	Cylinder and taper, finish processing	100,000 01
29	Internal grinding machine	Hole, finish processing	000100 01
30	Surface grinding machine	Plane, finish processing	010000 01
31	Planning machine 2	Plane, large-size part, finish processing	010000 11
32	Broaching machine	Plane, curved surface, finish processing	010010 01

The algorithm was implemented in C +  + . The population sizes of the internal and external iterations were set to 40 and 20, respectively. The number of evolutionary generations of the hybrid algorithm was set to 100, and the crossover and variation rates were set to 0.8 and 0.1, respectively.

**Figure 4:**
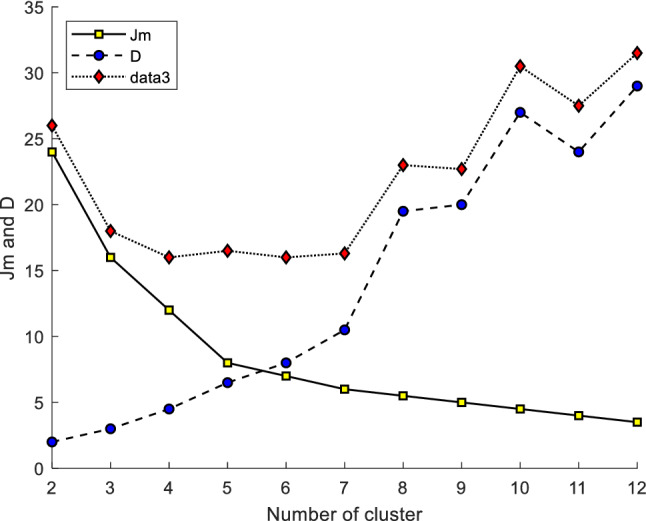
Relationship between *J*_*m*_, D, and the number of clusters.

**Figure 5:**
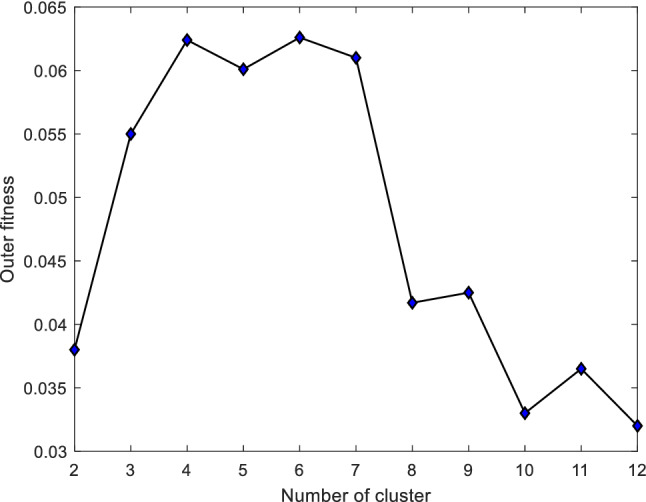
Relationship between the outer fitness and the number of clusters.

The variation of fuzzy clustering objective function *J*_*m*_ and the mean distance D between the cluster centers with respect to the number of clusters is shown in Fig. 4. *J*_*m*_ decreased monotonically with an increase in the number of clusters. D monotonically increased in the range (2, 10) (11, 12) and monotonically decreased in the range (10, 11). The sum of *J*_*m*_ and *D* was minimal when the number of clusters was 6. The variation of the outer iterative fitness function is shown in Fig. 5, and the maximum value of fitness was obtained when the number of clusters was 6. According to the optimal number (6) of the proposed algorithm, the optimal classification was obtained according to the principle of maximum affiliation. The optimal classification of manufacturing resources is shown in Table 2.

**Table 2:** Classification result of manufacturing resources.

Number of cluster	Machining equipment	Feature
1	Lathe 2, Lathe 3, Milling and drilling machine, Milling machine 5, Broaching machine	Cylinder and taper, plane, groove, hole, curved surface, step
6, 7, 12, 22, 32		
2	Drilling machine 1–5, Lathe 1, Lathe 5, Horizontal fine-boring machine, Cylindrical grinding machine,	Cylinder and taper, hole
2, 3, 4, 5, 9, 11, 13, 17, 29		
3	Vertical milling machine, Milling machine 1, Milling machine 3, Milling machine 4, Planning machine 1, Surface grinding machine	Plane, groove, step, finish processing
1, 18, 20, 21, 27, 30		
4	Milling machine 2, Milling machine 6–8, Planning machine 2	Plane, curved surface, large-size part, finish processing
19, 23, 24, 25, 31		
5	Lathe 4, Cylindrical grinder	Cylinder and taper, hole, finish processing
8, 28		
6	Lathe 6, Boring-milling machine 1–3, Coordinate setting boring machine	Cylinder and taper, plane, groove, hole, large-size part, finish processing
10, 14, 15, 16, 26		

Each manufacturing resource belongs to only one class, but each feature can belong to multiple classes. The second and fifth groups could process cylinder, taper, and hole features for small- and medium-sized parts, but the equipment in the fifth group could be used for finishing; thus, the two groups were divided into different groups. The first and sixth groups could handle cylindrical and conical, hole, plane, and groove features, but the equipment in the sixth group could handle large parts of these features and was thus used for finish machining. These equipment were in different partition groups although they could handle the same features. The main component of manufacturability evaluation is to evaluate whether each feature of the part has the corresponding processing equipment. By classifying processing equipment in groups, only the group with the evaluated feature needs to be searched. Therefore, the search time and space for processing equipment corresponding to the features is reduced and the efficiency of manufacturability evaluation is improved.

After finding the processing equipment corresponding to the features, the information model of the equipment needs to be established and used to evaluate whether the processing capability of the equipment meets the design requirements. This study used the object-oriented approach to build the model.

## Information modeling of manufacturing resources based on the object-oriented method

### Object-oriented method in the manufacturing resource model

The basic principle of the object-oriented approach is to identify and define entities in the objective world. Object-oriented methods have effective structural features, including classification, encapsulation, and inheritance, but it is a vague analytical model. Unlike the structural modeling approach, the object-oriented modeling approach emphasizes the relationships and states among objects during system measurement. The state of each object in the system is expressed through properties in the object-oriented modeling approach, and relationships and interactions between the objects are measured through events and messages. The structure of the object model can be described by objects, attributes, and associations^34^.

### Demands and structure of the manufacturing resource model based on the object-oriented method

A few factors such as the manufacturing capacity of enterprise resources, processing materials, and the processing capability for equipment processing (machining precision, working range, and the carrying capacity of the table) need to be considered during manufacturability evaluation. The manufacturability of a part relates not only to the processing equipment but also to the technological equipment, such as the cutting tool, fixture, and measuring tool. Thus, a detailed information model of manufacturing resources, which includes processing equipment and technological equipment, should be built to evaluate their processing capabilities. In the manufacturing resources model based on features, the manufacturing features are involved in this model too. Manufacturability evaluation not only assesses whether a part can be machined by the existing manufacturing resources but also selects the optimum processing equipment based on the different needs of the clients. The selection of equipment involves plenty of information, such as the state of equipment, processing cost, processing time, and location. When the information of the model is more detailed, the evaluation results are better. Thus, the model should contain as much information as much as possible and should be changed whenever necessary.

To meet the demands of manufacturability evaluation, the information model of manufacturing resources should be dynamic, integrated, and steady. The data of the manufacturing resources model should be stored in a coherent and safe way, and the data structure of the model should be convenient for data processing. The object-oriented class hierarchical structure model is constructed by taking advantage of encapsulation and inheritance of the object-oriented method to abstract manufacturing resources. Each class has its own subclasses.

**Figure 6:**
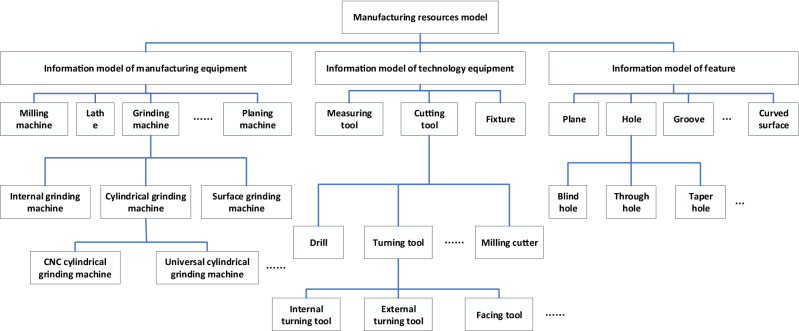
Manufacturing resources model.

According to the content of manufacturability evaluation, the manufacturing resources model comprises the information model of the manufacturing equipment class, technological equipment class, and feature class. The information model of the manufacturing equipment class mainly includes the processing equipment, which includes equipment such as the milling machine, grinding machine, and lathe. The grinding machine involves the cylindrical grinding machine, internal grinding machine, and surface grinding machine. The cylindrical grinding machine comprises equipment such as the CNC cylindrical grinding machine and universal cylindrical grinding machine. The information model of the technological equipment class includes cutting tools, measuring tools, and fixtures, and the information model of the feature class involves features that can be processed by the equipment in this manufacturing resources model, such as plane, hole, and groove. A schematic illustration is shown in Fig. 6.

**Figure 7:**
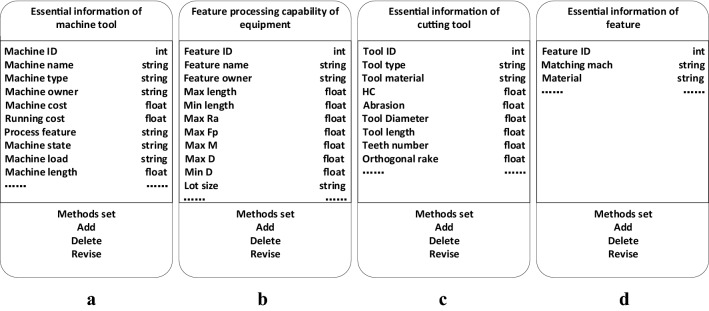
Object-oriented information model. (**a**) essential information of machine tool; (**b**) feature processing capability of the equipment; (**c**) essential information of cutting tool; and (**d**) essential information of the features.

The object-oriented information model of processing equipment contains two sections: the essential information model of specific processing equipment and processing capability information of processing equipment. The essential information model describes public information unrelated to processing. This information includes parameters such as the machine ID, machine name, machine type, machine owner, machine cost, and machine load. When a specific machine tool object belonging to a class of machine tools is built, a value should be assigned to these attributes. The essential information model of machine tools is depicted in Fig. 7(a). The processing capability model describes the capability of generating manufacturing features. The information includes aspects such as the feature ID, feature name, feature owner, max length (max length machined), min length (min length machined), max Ra (max roughness), max Fp (max form and position accuracy), max D (max diameter machined), min D (min diameter machined), and lot size. The feature processing capability model of the equipment is shown in Fig. 7(b).

The essential information model of the cutting tool and the features are shown in Figs. 7(c) and 7(d).

### Manufacturability evaluation based on manufacturing resources constraints

**Figure 8:**
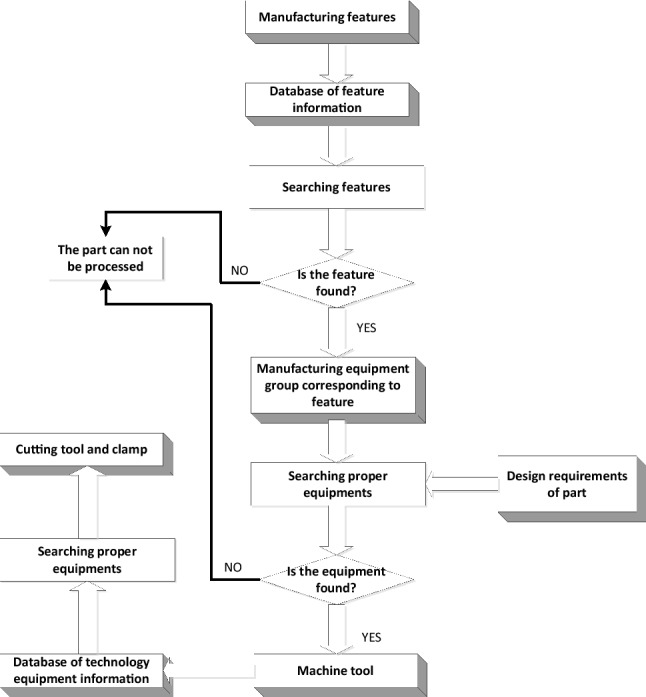
Manufacturability evaluation based on manufacturing resources constraints.

The manufacturability of a part is the extent to which a part can be adapted to suit the available manufacturing resources. This involves factors such as the machining cost, machining time, machining technology, and assembly process^35^. Based on this information, the manufacturing resources model based on the object-oriented method was built. An important step in manufacturability evaluation based on manufacturing resource constraints is to test whether the part and design features can satisfy the constraints and be machined by the existing manufacturing resources. Manufacturability evaluation is also based on feature and evaluation processes and is shown in Fig. 8.

- Step 1. The manufacturing feature is defined as the input. Thereafter, search the database for the feature information. If the feature is found, the part can be processed; otherwise, the part cannot be processed.
- Step 2. Search the machining tool group corresponding to the features according to the design requirements of the part. If the equipment that can process the feature is found, the part can be processed; otherwise, the part cannot be processed by the limited manufacturing resource.
- Step 3. Search the database for the technology equipment information. If the proper cutting tools and fixtures are found based on the design requirements, the part can be processed in the existing manufacturing environment; otherwise, the part cannot be processed.

There are two aspects to manufacturability evaluation: one is to test whether the part can be processed by the existing manufacturing resource and the other is to decide how the part can be processed effectively at a low cost. The first step is concerned only with the manufacturing resources. The next step involves finding an optimal way to process the part based on varying customer requirements.

## Conclusions

This study developed a hybrid algorithm comprising the GA and the FCM to group processing equipment according to the manufacturing and geometric features that can be processed by the equipment. The fuzzy rules employed could cope with the problem of the difference arising between the processing capabilities of modern processing equipment. The algorithm was tested on a variety of applications. The mathematical model of this algorithm was constructed, and the algorithm was tested with 32 processing machines. The results showed that the search space and search time of the processing equipment were successfully reduced by using the hybrid algorithm, indicating that it was reasonably effective and insensitive to the initial values.

The information model of manufacturing resources was built by using the object-oriented method based on features. The features were also involved in this model. The model could provide information required for product development and manufacturability evaluation to determine whether the product could be processed by employing the existing manufacturing resource. The information model could also yield information for computer-aided process planning and render the manufacturing resources conducive for effective management. The framework of manufacturability evaluation based on the constraints of the proposed manufacturing resources model was defined. By adopting this framework, the time taken for evaluation could be reduced. The manufacturing resources model proved beneficial in enhancing the overall performance of the company, resulting in improved and feasible decision-making among the management. The model can be further refined in the future by introducing additional manufacturing characteristics to make the information more detailed and the manufacturability assessment and decision-making more effective and feasible.

## Data Availability

All data generated or analyzed during this study are included in this published article.
